# Upfront frameless hypofractionated gamma knife radiosurgery for large posterior Fossa metastases

**DOI:** 10.1007/s10143-025-03572-4

**Published:** 2025-05-15

**Authors:** Yavuz Samanci, Serhat Aydin, Ali Haluk Düzkalir, M. Orbay Askeroglu, Selcuk Peker

**Affiliations:** 1 Department of Neurosurgery, Koç University School of Medicine, Türkiye Davutpasa Caddesi No:4, Zeytinburnu/İstanbul, 34010 Türkiye; 2 Department of Neurosurgery, Gamma Knife Center, Koç University Hospital, Istanbul, Türkiye; 3 Koç University School of Medicine, Istanbul, Türkiye; 4 Department of Neurosurgery, Koç University Hospital, Istanbul, Türkiye

**Keywords:** Hypofractionation, Gamma knife radiosurgery, Metastatic brain tumors, Posterior fossa, Upfront radiosurgery

## Abstract

The management of large metastatic brain tumors (METs), particularly those in the posterior fossa (pf-METs), is challenging. While surgery can alleviate symptoms, it carries the risk of complications such as leptomeningeal disease (LMD). Upfront hypofractionated Gamma Knife radiosurgery (hf-GKRS) has shown promise as an alternative approach for managing large METs. This study assesses the efficacy and safety of upfront hf-GKRS for treatment-naïve large pf-METs. In this retrospective, single-center study, 40 patients with 42 pf-METs received hf-GKRS from October 2017 to June 2024. Patients eligible for the study were 18 years or older, had histologically confirmed malignancy, large pf-METs (> 4 cm^3^), and a minimum of two follow-up MRI scans. The primary outcome was local control (LC), with secondary assessments of distant intracranial failure (DICF), intracranial progression-free survival (PFS), overall survival (OS), and toxicity. LC was achieved in 88.1% of pf-METs over a median follow-up of 6 months (mean: 13.7 months). LC rates at 6, 12, and 24 months were 95.8%, 95.8%, and 74.5%, respectively. Local failure (LF) occurred in 11.9% of cases, with a median recurrence time of 12 months. DICF was noted in 35% of patients, while no cases of LMD were reported. Intracranial PFS rates at 6, 12, and 24 months were 54.1%, 39.0%, and 16.7%, respectively, with a median PFS of 8 months. Symptomatic hydrocephalus developed in one patient (2.5%). Controlled primary tumor status (HR: 0.17, *p* = 0.036) was significantly associated with lower risk of death, while no other parameters were predictive of LC, DICF, or intracranial PFS. hf-GKRS demonstrates strong efficacy and safety as a primary treatment for selected, treatment-naïve large pf-METs over a relatively short follow-up duration. Further studies are warranted to refine patient selection, fractionation, and dosing strategies for this challenging population.

## Introduction

Approximately one-third of cancer patients develop metastatic brain tumors (METs), with incidence increasing due to advancements in systemic therapies that prolong survival [33]. Among these, approximately 20% occur in the posterior fossa (pf-MET), where surgical intervention is traditionally employed to alleviate symptoms, particularly those associated with mass effect caused by large pf-METs. However, achieving durable local control (LC) necessitates postoperative radiotherapy (RT) [49]. Additionally, posterior fossa surgery carries a risk of further metastatic dissemination or leptomeningeal disease (LMD), potentially compromising the efficacy of subsequent RT [45].

Although the efficacy of stereotactic radiosurgery (SRS), especially hypofractionated SRS (hf-SRS), has been well-documented for large METs in other locations [16, 18], few studies have specifically addressed only staged SRS (ss-SRS) applications to large pf-METs [9, 23, 50]. Unlike ss-SRS, which requires a 30-day interval for repair as suggested by Angelov et al. [2] and complicates compliance as shown by Lo et al. [23], daily hf-SRS offers a more practical and effective approach for managing large pf-METs. Hypofractionation also allows for lower doses to adjacent healthy tissues, further offering a biologically advantageous approach [19].

This single-center study aims to assess specifically the efficacy and safety of upfront, frameless hypofractionated Gamma Knife radiosurgery (hf-GKRS) for treatment-naïve large (> 4 cm³) pf-METs.

## Materials and methods

This retrospective study was approved by the Institutional Review Board of Koç University (Approval No: 2022.022.IRB1.017). All subjects consented to participate prior to their inclusion.

This study included 40 patients (42 pf-METs) who underwent hf-GKRS between October 2017 and June 2024. Eligible patients were ≥ 18 years old, had histologically confirmed malignancy, large pf-METs (> 4 cm³), and at least two follow-up MRI scans. Patients with prior surgical resection or irradiation of the target pf-MET were excluded. Patients with complete fourth ventricle effacement were also excluded due to the risk of acute obstructive hydrocephalus. Cases of partial effacement were approached cautiously, particularly in asymptomatic patients. Patients with radiological evidence of hydrocephalus were excluded unless they had undergone prior cerebrospinal fluid (CSF) diversion.

hf-GKRS was selected based on various factors, including medical inoperability, multiple METs, or patient preference. Surgical ineligibility was primarily determined by poor functional status (Karnofsky Performance Scale [KPS] < 70) or extensive systemic disease burden. Patient classification was conducted using the RTOG Recursive Partitioning Analysis (RPA) [8] and Graded Prognostic Assessment (GPA) [44] scales, with documentation of concurrent systemic therapies, including chemotherapy, targeted therapy, and/or immunotherapy [13]. While RPA remains a widely used prognostic tool, GPA provides a more refined stratification, particularly across different primary malignancies. Due to the heterogeneity of our cohort, diagnosis-specific GPA (ds-GPA) stratifications were not incorporated. All patients had histopathological confirmation of their primary malignancy; however, specific mutational or receptor status (e.g., EGFR, ALK, KRAS for lung cancer; HER2, ER/PR for breast cancer) was not included in this analysis.

hf-GKRS was performed using the Leksell Gamma Knife^®^ Icon™ (Elekta Instrument AB, Stockholm, Sweden), with detailed methodology previously described [40]. In the absence of established guidelines for optimal treatment protocols, hf-GKRS regimens were determined based on tumor characteristics, patient-related factors, and dose constraints. The effectiveness of different dosing regimens was assessed by calculating the equivalent dose in 2 Gy fractions (EQD2) with an α/β ratio of 10 [48]. Dose metrics for normal brain tissue (V18, V21, V24 for 3-fraction regimens and V25, V28.8, V30 for 5-fraction regimens) and brainstem tissue (V15.9 for 3-fraction, V23 for 5-fraction, and Dmax for both) were computed [1, 10, 29, 30, 47]. Treatment was delivered on consecutive days without an additional margin. All patients received dexamethasone during SRS, and tapering was initiated 1–2 weeks post-GKRS.

Routine clinical assessments and MRI scans were conducted 1–2 months after initial hf-GKRS, followed by subsequent evaluations every 2–4 months based on imaging and clinical findings. Radiological assessment was performed using the Response Assessment in Neuro-Oncology Brain Metastases criteria, which categorize treatment response as follows: complete response (CR), partial response (PR) (≥ 30% reduction in lesion size), progressive disease (PD) (≥ 20% increase in lesion size), or stable disease (SD) in cases not meeting CR, PR, or PD criteria [22]. The primary outcome was the LC rate, defined as the proportion of lesions achieving CR, PR, or SD, monitored until local failure (LF) or death. Intracranial progression-free survival (PFS) was defined as the time to LF, distant intracranial failure (DICF), or death, while overall survival (OS) was defined as the time from treatment to last follow-up or death. Adverse events were classified according to the Common Terminology Criteria for Adverse Events version 5.0 [5].

### Statistical analysis

Statistical analyses were performed using SPSS 29.0 (Statistical Package for the Social Sciences, SPSS Inc., Chicago, IL, USA) and Rstudio 2023.09.1 Build 494. All tests were two-sided, with statistical significance set at *p* < 0.05. Analyses of LC and LF were conducted on a per-tumor basis, while OS, PFS, and DICF were assessed on a per-patient basis. LC was analyzed using the Fine-Gray proportional hazards model, treating death as a competing event. PFS and OS were analyzed using Kaplan-Meier estimates and multivariable Cox proportional hazards models. Clinically relevant variables with a p-value < 0.2 in univariate analysis were included in the multivariate model. Hazard ratios (HRs) with 95% confidence intervals (CIs) were computed to assess the impact of these variables on survival outcomes.

## Results

Table 1 outlines the baseline characteristics and clinical features of the cohort. The majority of patients were male (60%), with a median age of 63.5 years. Lung cancer was the most common primary malignancy (37.5%). The median time from initial diagnosis to intracranial metastasis was 26 months. The cerebellum was the most frequent site of pf-METs (95.2%), and 50% of patients had multiple intracranial METs (range: 2–14). Headache was the most common presenting symptom (30%), while 57.5% of patients exhibited no neurological deficits. Most patients had good functional status, with a median KPS score of 90. The majority were classified as RPA class II (67.5%), with a GPA score of 2 being the most common (32.5%). Seven patients (17.5%) had a history of non-target SRS, and six patients were receiving concurrent systemic therapy.

**Table 1:** Baseline characteristics and clinical features of the study cohort

Parameters	Value
Number of patients	40
Number of lesions (total)	42
Sex ratio (female/male)	2:3
Median age (range), years Number of patients aged ≥ 65 years, n (%)	63.5 (26–83) 16
The primary site, n (%) Lung Breast Gastrointestinal Genitourinary Other	15 (37.5) 8 (20) 7 (17.5) 7 (17.5) 3 (7.5)
Median time from diagnosis to intracranial metastasis (range), months	26 (0-209)
Location of metastatic brain tumors, n (%) Cerebellum Brainstem	40 (95.2) 2 (4.8)
Presenting symptoms, n (%) Headache Imbalance Incidental Paresis Dizziness Other	12 (30) 10 (25) 8 (20) 6 (15) 3 (7.5) 1 (2.5)
Median Karnofsky performance status (range) Number of patients with score ≤ 70, n (%)	90 (50–90) 7 (17.5)
Uncontrolled primary cancer, n (%)	14 (35)
Extracranial metastasis, n (%)	22 (55)
Number of metastatic brain tumors, n (%) 1 2–5 6–10 > 10	20 (50) 13 (32.5) 5 (12.5) 2 (5)
Recursive partitioning analysis classification, n (%) Class I Class II Class III	8 (20) 27 (67.5) 5 (12.5)
Graded prognostic assessment scoring, n (%) ≤ 1 1.5–2.5 ≥ 3	5 (12.5) 26 (65) 9 (22.5)
History of non-target stereotactic radiosurgery, n (%)	7 (17.5)
Concurrent chemotherapy, targeted therapy, and/or immunotherapy), n (%)	6 (15)
Median follow-up time (range), months	6 (4–51)

Table 2 summarizes the radiosurgical data. The most common reason for hf-GKRS referral was patient preference (37.5%). The median interval between metastasis diagnosis and hf-GKRS was one week, with a median treatment volume of 8.1 cm³. Four distinct dosing regimens were employed: 24 Gy/3 fractions in nine patients (range: 4.2–13 cm³), 27 Gy/3 fractions in seven patients (range: 4.1–15 cm³), 25 Gy/5 fractions in two patients (range: 8.4–15 cm³), and 30 Gy/5 fractions in 24 pf-METs (range: 4.1–17.7 cm³). The EQD2_10_ values for these regimens were 36 Gy, 42.75 Gy, 31.25 Gy, and 37.5 Gy, respectively. The median dose metrics for normal brain tissue were as follows: 22.3 cm³ at V18, 17.95 cm³ at V21, and 14.85 cm³ at V24 (3-fraction); and 18.95 cm³ at V25, 15.5 cm³ at V28.8, and 14.15 cm³ at V30 (5-fraction). The median brainstem dose was 0 cm³ for both V15.9 in 3-fraction and V23 in 5-fraction treatments. The brainstem median Dmax was 10.65 Gy for 3-fraction and 12.35 Gy for 5-fraction (Table 3).

**Table 2:** Baseline radiosurgical features of the study cohort

Parameters	Value
Reason for patient referral, n (%) Patient preference Surgical risk Multiple metastases Low Karnofsky performance status (KPS < 70)	15 (37.5) 10 (25) 10 (25) 5 (12.5)
Median time from metastasis to hf-GKRS (range), weeks	1 (0–9)
Median tumor volume (range), cm^3^	8.1 (4.1–17.7)
Median cumulative tumor volume (range), cm^3^	10.4 (4.2–26.6)
Dose regimens, n (%) 5 × 6 Gy 3 × 8 Gy 3 × 9 Gy 5 × 5 Gy	24 (57.1) 9 (21.4) 7 (16.7) 2 (4.8)
Median isodose line, (range), %	45 (40–60)
Median maximum dose to the margin (range), Gy	60 (45–75)
Median EQD2_10_ (range)	40 (31.25–42.75)

hf-GKRS: hypofractionated Gamma Knife radiosurgery, EQD2: Equivalent dose in 2 Gy fractions

**Table 3:** Median dose metrics for normal brain tissue and brainstem of the 3-fraction and 5-fraction gamma knife radiosurgery

Parameters	Value (range)
3-fraction V18, cm³ V21, cm³ V24, cm³ Brainstem V15.9, cm³ Brainstem Dmax, Gy 5-fraction V25, cm³ V28.8, cm³ V30, cm³ Brainstem V23, cm³ Brainstem Dmax, Gy	22.3 (8.6–52.6) 17.95 (7-42.4) 14.85 (5.7–34.8) 0 (0-1.7) 10.65 (2.8–38.2) 18.95 (7.4–65.2) 15.5 (6.2–52.2) 14.15 (5.8–47.5) 0 (0-9.5) 12.35 (3.5–66.6)

**Fig. 1:**
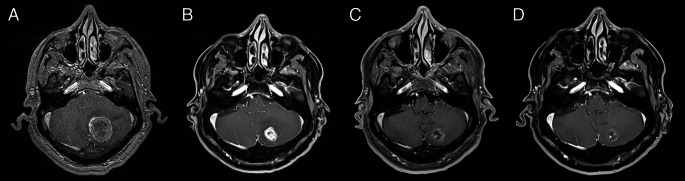
An illustrative case of hypofractionated Gamma Knife radiosurgery. A 60-year-old male with a history of colon cancer presented with dizziness and was found to have a posterior fossa metastasis (9.7 cm^3^). The patient was referred to radiosurgery for surgical risks, and the lesion was treated with a marginal dose of 30 Gy in 5 fractions at the 40% isodose line (**A**). Follow-up imaging at 2 months (**B**), 5 months (**C**), and 13 months (**D**) post-radiosurgery demonstrated local control. The patient remained alive at the time of analysis

The median follow-up duration was 6 months (range: 4–51 months, mean:13.7 months), with the relatively short follow-up primarily driven by patient-related factors, including high systemic disease burden and early mortality in some cases. The LC rate for the cohort was 88.1%. Neuroimaging revealed that 24 tumors (57.1%) exhibited PR, while 13 tumors (31%) remained stable. The estimated cumulative LC rates at 6, 12, and 24 months were 95.8% (95% CI: 73.9–99.4), 95.8% (95% CI: 73.9–99.4), and 74.5% (95% CI: 36.9–91.7), respectively. A representative case is illustrated in Fig. 1.

LF occurred in 5 pf-METs (11.9%), with a median volume of 8.6 cm³ (range: 5.5–15 cm³) and a median onset of 12 months (range: 4–24 months). These pf-METs underwent repeat SRS, with four (80%) showing regression after the second procedure. However, in one case, surgical intervention was required 12 months after the second SRS due to persistent PD. DICF was observed in 14 patients (35%), with a median time to progression of 5.5 months (range: 1–33 months). Notably, none of these patients exhibited signs of LMD. The estimated intracranial PFS rates at 6, 12, and 24 months were 54.1%, 39.0%, and 16.7%, respectively, with a median PFS of 8 months (95% CI: 3.0–13.0).

**Fig. 2:**
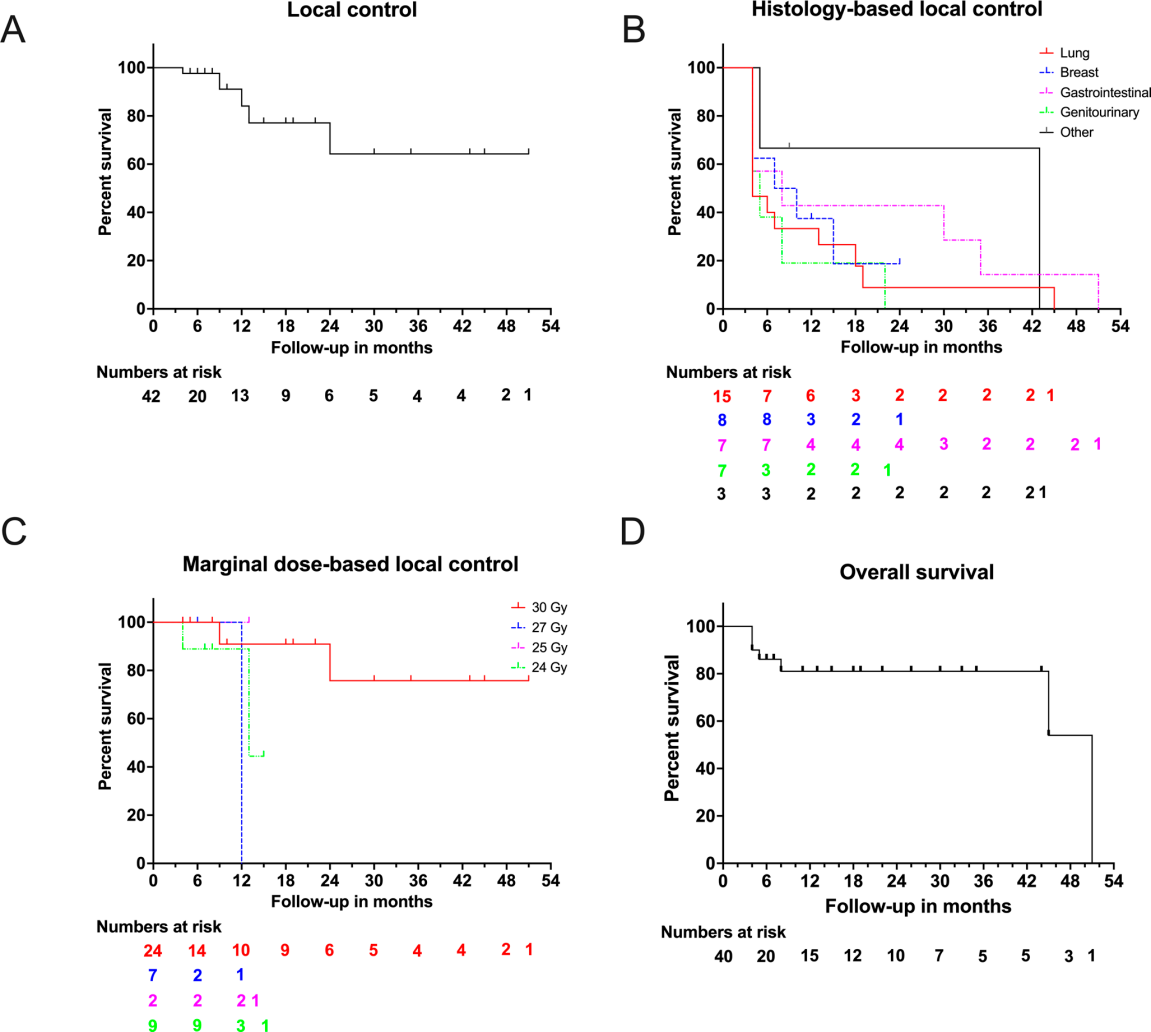
Local control (**A**), marginal dose-based local control (**B**), histology-based local control (**C**) and overall survival estimates (**D**) of the study cohort

A single case (2.5%) of grade 3 hydrocephalus was identified 6 weeks after hf-GKRS in a 63-year-old male with lung carcinoma, presenting with moderate headache and imbalance. This required ventriculoperitoneal shunting. As of the analysis date, 8 patients (20%) had died. Among these, only one patient (12.5%) succumbed to neurological causes due to extensive intracranial disease, while the remaining 87.5% (7 patients) died from systemic disease progression. Estimated OS rates at 6, 12, and 24 months were 58.5%, 46.0%, and 29.6%, respectively (Fig. 2).

In the multivariable Cox proportional hazards model for OS, controlled primary tumor status was the only variable significantly associated with improved survival. Patients with controlled primary tumors had a significantly lower risk of death (HR = 0.17, 95% CI: 0.03–0.89, *p* = 0.036). No other covariates reached statistical significance. In multivariable Fine-Gray regression analysis accounting for death as a competing event, none of the evaluated covariates—including primary histology (lung vs. others), lesion volume, total dose, maximum dose, or the use of adjuvant systemic therapy—were significantly associated with local control. The subdistribution hazard ratios ranged from 0.86 to 1.80, with all p-values > 0.2. This analysis demonstrated that the cumulative incidence of LF remained low during the early follow-up period but could not be reliably assessed beyond the median follow-up due to the high competing risk of death (Fig. 2; Table 4).

**Table 4:** Multivariable regression results for local control (LC) and overall survival (OS)

Covariate	LC – Univ. SHR (95% CI), *p* †	LC – Multiv. SHR (95% CI), *p*†	OS – Univ. HR (95% CI), *p*‡	OS – Multiv. HR (95% CI), *p*‡
Primary cancer type (lung cancer vs. others)	1.26 (0.28–5.67) 0.76	-	0.75 (0.15–3.88) 0.73	-
Treatment volume, cm^3^ (continuous)	1.11 (0.89–1.38) 0.35	-	N/A	-
Marginal dose (continuous)	0.86 (0.58–1.29) 0.47	-	N/A	-
Maximum dose (continuous)	1.00 (0.93–1.08) 0.98	-	N/A	-
Concurrent systemic chemotherapy/immunotherapy (yes)	1.80 (0.44–7.41) 0.42	-	2.53 (0.46–13.9) 0.29	-
Controlled primary tumor (yes)	-	-	*0.168 (0.03–0.89)* *0.036*	*0.168 (0.03–0.89)* *0.036*
Extracranial metastases (no)	-	-	51.66 (0.07-40156.37) 0.25	-
Number of metastatic brain tumors (single)	-	-	0.46 (0.09–2.41) 0.36	-

Univ.: Univariate, Multiv.: Multivariate, SHR = Subdistribution hazard ratio, HR: hazard ratio, CI: confidence interval

†Fine-Gray competing risks regression (death as competing risk)

‡Cox proportional hazards regression for overall survival

## Discussion

This is the first study to specifically evaluate the efficacy and safety of frameless hf-GKRS for treating treatment-naïve large pf-METs, a particularly challenging subset of METs due to the complex anatomy of the posterior fossa and the risks associated with mass effect. Our findings demonstrated high LC rates, with 95.8% at 6 and 12 months and 74.5% at 24 months, comparable to outcomes achieved with other radiosurgical or surgical approaches. These results support hf-GKRS as a viable treatment option for patients with large pf-METs who are either ineligible for surgery or decline surgical intervention. However, it should also be noted that our study cohort was composed exclusively of treatment-naïve patients with relatively preserved neurological function (median KPS: 90), which reflects a highly selected subgroup of pf-MET patients. This design choice allowed for a more uniform assessment of hf-GKRS efficacy in the absence of confounding effects from prior local therapies. However, it also limits the generalizability of our findings to the broader pf-MET population, many of whom may present with symptomatic hydrocephalus, brainstem compression, or have undergone prior surgical resection. These patients often exhibit lower performance status and may require more urgent surgical intervention. Therefore, while our results suggest that upfront hf-GKRS is both safe and effective in well-selected, stable candidates, caution is warranted when extrapolating these outcomes to more complex or symptomatic cases. Also, while our findings suggest that short-term LC following hf-GKRS is favorable in patients with large pf-METs, this interpretation should be made with caution. Due to the limited follow-up duration and the high incidence of systemic disease progression, long-term durability of LC remains uncertain. Standard Kaplan-Meier methods tend to overestimate LC by treating death as a censoring event rather than as a competing risk. Therefore, we performed a competing risks analysis using the Fine-Gray model, which offers a more conservative and clinically realistic estimate of LC in the presence of competing events such as death. The results reaffirm that while early LF is uncommon, conclusions regarding long-term control should remain tentative in this cohort. Future studies with larger sample sizes and longer follow-up durations are needed to more definitively characterize long-term LC in this population.

## Surgery vs. Radiosurgery for posterior Fossa metastases

Selecting the appropriate treatment for a pf-MET requires a comprehensive assessment of factors including primary cancer type, tumor size, clinical condition, and extent of systemic disease. Neurosurgeons and oncologists exercise particular caution with pf-METs due to the risk of acute obstructive hydrocephalus, which, if not promptly managed, can rapidly progress to coma and death. Consequently, there is a trend toward emergent surgical resection for these tumors. However, surgical intervention has also notable disadvantages, including its invasiveness, prolonged recovery time, interruption of systemic therapy, and an increased risk of complications such as LMD, CSF fistula, cerebellar mutism syndrome, and infections [7, 17, 42, 45]. Among these risks, LMD is particularly concerning, as affected patients have a median survival of only 2–4 months [15, 45].

Several studies have reported that infratentorial METs are more strongly associated with LMD following resection compared to supratentorial tumors [35, 37]. Siomin et al. [43] found that LMD was significantly more frequent following surgical resection than after RT for pf-METs (50% vs. 6.5%). However, OS did not significantly differ between the RT and surgery groups in that study, suggesting that while SRS may reduce LMD risk, its impact on OS is likely confounded by systemic disease progression. Our findings align with these results, as no cases of LMD were observed in our cohort, highlighting the potential of upfront hf-GKRS to substantially reduce LMD incidence compared to surgery. While upfront SRS may lower LMD risk, most centers now routinely administer SRS to the resection cavity following surgery [26, 41]. A recent study particularly analyzed 64 patients with large pf-METs (≥ 4 cc) treated with SRS or surgery followed by SRS (S + SRS) from 2009 to 2020 [14]. Patients in the S + SRS group had more severe symptoms, larger lesion volumes (29.8 cm³ vs. 6.7 cm³, *p* < 0.001), higher rates of fourth-ventricle compression (96% vs. 47%, *p* < 0.001), and hydrocephalus (29% vs. 0%, *p* < 0.001) compared to the SRS group. The S + SRS group also had a higher GPA and better OS of 26 months versus 12 months for SRS (*p* = 0.001). While LF rates were similar (17 vs. 12 months), the findings suggest that S + SRS should be seriously considered for patients with large PF-BrM. Although postoperative SRS appears to be an effective and safe adjuvant therapy, pre-SRS offers potential advantages, including a lower risk of radiation necrosis (RN) and LMD, improved tumor targeting, and reduced radiation exposure to healthy tissue.

Another advantage of SRS is its ability to treat multiple lesions, including deep-seated METs. In a prospective observational study, Yamamoto et al. [52] compared SRS outcomes in patients with 2–4 METs vs. those with 5–10 METs and found no significant difference in OS or adverse event rates. Another study comparing SRS with whole-brain radiotherapy in patients with 4–15 METs demonstrated superior neurocognitive function, increased OS, and similar LC [21]. These findings support the use of SRS for up to 15 METs, with many centers routinely and safely treating even higher numbers. Consistent with these reports, 50% of patients in our cohort had multiple METs (range: 2–14) alongside a pf-MET, making them ineligible for surgery but suitable for SRS. LF occurred in only two of these patients (10%), further supporting the role of hf-GKRS in this subset of patients.

## Hypofractionated stereotactic radiosurgery

A key aspect of this study was the use of hypofractionation, which optimizes LC while minimizing toxicity to surrounding tissues. This is particularly advantageous for large pf-METs, where single-fraction (sf-SRS) requires high radiation doses that might pose increased risks. According to the RTOG-9005 safety guidelines for sf-SRS, lesions measuring 3.1 to 4.0 cm in diameter (approximately 14 to 33.5 cm³) are restricted to a maximum dose of 15 Gy, a dose linked to suboptimal tumor control. For lesions exceeding 4 cm in diameter, there is currently no standardized single-fraction dosing recommendation [32]. In addition to limited LC, sf-SRS for large METs is also associated with an increased risk of treatment-related complications. sf-SRS carries a notable risk of RN, which may occur one to two years post-treatment and can range from asymptomatic radiographic findings to severe symptoms such as headache, lethargy, seizures, or even death. Reported rates of RN with sf-SRS vary between 13 and 30% [46], and sf-SRS in METs with V12 volumes of 5 cc, 10 cc, or > 15 cc resulted in RN risks of approximately 10%, 15%, and 20%, respectively, suggesting that sf-SRS may not provide an acceptable safety profile for larger METs [28]. A retrospective study evaluated 49 patients with 51 large pf-METS (median tumor volume: 4.96 cm³) treated with sf-GKRS [34]. At the first follow-up (2 months posttreatment), tumor volume decreased by 58.66%. The LC rate was 98.1%, and median OS was 8.36 months. During follow-up, 6.1% (3 patients) developed symptomatic RN: one required laser interstitial thermal therapy (14 months post-GKRS), another was treated with bevacizumab and needed a ventriculoperitoneal shunt (23 months post-GKRS), and the third was managed with steroids alone (15 months post-GKRS). Additionally, two patients had asymptomatic RN requiring no treatment. While RN remains a well-recognized complication of radiosurgery, potential injury to the brainstem must also be carefully considered, particularly in posterior fossa lesions where the therapeutic margin is inherently limited. Prior studies have highlighted the risk of brainstem injury when dose constraints are exceeded, especially in lesions larger than 4–5 cm³ or when treating with single-fraction regimens delivering ≥ 15 Gy marginal doses [6, 24, 39]. Although no cases of brainstem toxicity were observed in our cohort, likely due to adherence to dose-volume thresholds and relatively shorter follow-up, this does not preclude the potential for delayed complications such as cranial neuropathies or symptomatic edema.

An alternative to sf-SRS is staged SRS (ss-SRS), which delivers a higher total dose over two or three sessions [9, 23, 50]. Unlike daily hf-SRS, ss-SRS aims to reduce toxicity by extending the interval between fractions, leveraging the typical tumor volume reduction observed between stages. This strategy exposes a smaller brain volume to radiation in later sessions, potentially lowering toxicity. Angelov et al. [2] suggested that the higher per-session doses used in ss-SRS may enhance tumor cell death compared to hf-SRS, where doses typically range from 6 to 9 Gy. However, no significant association was found between fractionation dose and improved LC. Several studies have evaluated the outcomes of ss-SRS in patients with large pf-METs. Hori et al. [9] first reported outcomes for ss-SRS in 21 patients with large pf-METs (median volume: 7.43 cm³), achieving a 1-year LC rate of 83% with no post-treatment complications. Wang et al. [50] analyzed 40 patients with 45 large pf-METs (mean volume: 12.3 cm³) treated with ss-SRS, with median doses of 12.5 Gy per stage. Their LC rates at 6, 12, and 24 months were 97.5%, 86.0%, and 62.2%, respectively, with RN in 17.5% of patients. Lo et al. [23] studied four patients with large pf-METs (median volume: 25.6 cm³) who received ss-SRS at doses of 10–13 Gy, reporting no tumor progression and no radiation-induced complications. One handicap of this approach is the recommended 30-day interval between stages, as suggested by Angelov et al. [2], to allow time for repair of late radiation effects. This prolonged interval complicates patient compliance, as observed in the study by Lo et al. [23], where one of four patients was unable to complete the treatment due to clinical deterioration before the second stage. Given these challenges, daily hf-SRS appears to be a more practical and feasible approach for managing large pf-METs while maintaining treatment efficacy and reducing logistical barriers for patients.

### The role of hf-SRS in large METs and pf-METs

Since Wiggenraad et al. [51] introduced hf-SRS for large METs, multiple single-institution studies have demonstrated its ability to achieve equal or superior LC while reducing RN, even in larger volumes [4, 11, 12, 25, 31, 36, 40]. Redmond et al. [38] pooled data from 56 studies, showing that sf-SRS provided a 1-year LC rate of 69–75% for lesions 2.1–4 cm in size, whereas hf-SRS increased the 1-year LC rate to approximately 80%. A meta-analysis of 1,887 METs compared sf-SRS and hf-SRS in lesions 4–14 cm³ (group A) and > 14 cm³ (group B) [18]. In group A, 1-year LC rates were 77.6% for sf-SRS and 92.9% for hf-SRS, while in group B, LC rates were 77.1% and 79.2%, respectively. RN rates were significantly lower with hf-SRS in smaller METs (23.1% vs. 7.3%, *p* = 0.003) and trended lower in larger METs (11.7% vs. 6.5%, *p* = 0.29).

While hf-SRS has been widely adopted as an upfront or post-surgical treatment for large METs, data on its application specifically for pf-METs remain limited [20, 32]. Matsunaga et al. [27] analyzed a mixed cohort of 27 patients who underwent GKRS (single, 2-, 3-, or 5-fraction regimens) for pf-METs (median volume: 18.3 cm³, range: 11.3–27.2 cm³). They reported cumulative LC rates of 91.7% at 3 months, 70.8% at 6 months, and 64.4% at 12 months, with 4 patients (14.8%) experiencing adverse events at a median of 9 months. The cumulative incidence of symptomatic radiation injury was 0.0% at 6 months, 16.7% at 9 months, and 24.2% at 12 months post-GKRS. In our cohort (tumor volumes: 4.1–17.7 cm³), the estimated cumulative LC rates at 6, 12, and 24 months were 95.8%, 95.8%, and 74.5%, respectively, with no cases of RN. This absence of RN may be attributed to our strict adherence to dose constraints for normal brain and brainstem tissues [1, 10, 29, 30, 47]. Additionally, concurrent systemic therapy may influence RN risk. However, a systematic review by Borius et al. [3] found that most systemic agents do not significantly increase RN risk when administered alongside SRS. Evidence suggests that concurrent systemic therapy does not require a washout period, and delays in systemic therapy may even be harmful. In our cohort, 6 patients (15%) were receiving concurrent systemic therapy, and none developed RN. Furthermore, concurrent systemic treatment was not a significant predictor for LC, likely due to the low event rate in this subgroup. A key finding in our study was that only 1 of 8 deceased patients (12.5%) died from neurological causes, and this patient had multiple intracranial METs. The remaining 7 patients (87.5%) died from systemic disease progression, underscoring that OS in this cohort was primarily determined by extracranial disease burden rather than local treatment effects. Notably, no patients with isolated pf-METs died from neurological causes, reinforcing that hf-GKRS did not contribute to excess neurological mortality. These findings suggest that while hf-GKRS is highly effective for LC, OS remains largely dictated by systemic disease status, emphasizing the need for integrated oncological management in this patient population.

## Optimizing fractionation in hf-SRS

One of the key challenges in hf-SRS is determining the optimal fractionation dose, as no standardized regimen currently exists. The American Society for Radiation Oncology guidelines [49] recommend 27 Gy in 3 fractions or 30 Gy in 5 fractions for lesions 2–4 cm in size, and in our study, most lesions (57%) were treated with 30 Gy in 5 fractions. The variability in dosing regimens in our cohort reflects a tailored approach to hf-GKRS, balancing effective LC with minimizing toxicity risks. For larger tumors (up to 17.7 cm³), a conservative fractionation regimen of 30 Gy over 5 fractions was predominantly used to reduce RN risk. Conversely, smaller tumors (4.1–15 cm³) were treated with 24–27 Gy over 3 fractions, providing effective LC with fewer fractions. This adaptive dosing strategy in hf-GKRS allows for customization based on tumor volume while also considering patient-specific and tumor-related factors. Ultimately, dose selection depends on a combination of lesion size, location, histology, and total irradiated volume. Although we analyzed treatment outcomes across different hypofractionation regimens, our study lacked sufficient statistical power to detect significant differences between fractionation strategies. Given the small cohort size and variability in tumor volumes, we cannot draw definitive conclusions regarding the superiority of one fractionation regimen over another. Future prospective studies with larger sample sizes are warranted to further refine fractionation strategies and establish optimal dosing guidelines for pf-METs.

## Prognostic classification in radiosurgery outcomes

The selection of an appropriate prognostic classification system remains an important factor in radiosurgical outcomes. While the RTOG RPA classification is widely utilized, it is a relatively older system that may not fully account for the heterogeneity of METs from different primary malignancies. More recently, the GPA and ds-GPA models have emerged as refined prognostic tools, offering disease-specific stratifications. Although we incorporated the GPA scale in our study, ds-GPA stratifications were not analyzed due to heterogeneity within our patient cohort. Future studies could further explore the prognostic value of ds-GPA, particularly in its application to treatment outcomes in pf-METs, potentially providing more precise prognostic insights for patients undergoing hf-GKRS.

### Limitations

This study has several limitations. Its retrospective, single-center design may introduce selection bias and limit the generalizability of findings. Our cohort represents a highly, treatment-naïve selected patient population, with a median KPS of 90, indicating relatively preserved neurological function. This differs from many surgical series of pf-METs, where patients often present with lower KPS due to mass effect-related symptoms (e.g., hydrocephalus, brainstem compression). So, our findings may not be fully generalizable to all patients with pf-METs, particularly those with symptoms or previously treated or recurrent disease. While our median follow-up of 6 months was sufficient for assessing early LC and toxicity, it may not fully capture late adverse effects or delayed tumor progression. High censoring rates in survival analysis also weaken long-term outcome estimates, highlighting the need for larger cohorts with extended follow-up. Although prior studies have suggested that factors such as lesion volume or radiation dose may influence LC, our competing risks analysis did not identify any statistically significant predictors in this small cohort. This may reflect limited statistical power due to the low number of local failure events. However, the wide confidence intervals observed, particularly for systemic therapy and primary histology, indicate that further investigation in larger cohorts is warranted. While we reported LC rates across various dose regimens, the study was not statistically powered to determine whether one fractionation regimen was superior to another. Larger, multi-center studies are needed to validate optimal dose-fractionation strategies for pf-METs. A significant limitation of this study is the lack of a comparative control group, particularly patients undergoing surgical resection for large pf-METs. Given the substantial number of patients treated at our institution, a propensity-matched analysis could provide valuable insights by comparing hf-GKRS and surgical resection in similar patient populations. A further limitation is the absence of molecular characterization of metastatic lesions, particularly in patients with lung and breast cancer, which may have influenced treatment response.

Future research should aim to address the limitations of this retrospective study through prospective, multicenter trials with larger and more diverse patient populations. Such studies would allow for more robust comparisons between treatment modalities and help validate the efficacy and safety of hf-GKRS in broader clinical contexts. Additionally, biomarker-driven patient selection may enhance personalized treatment strategies by identifying subgroups more likely to benefit from SRS. Extended follow-up analyses are also needed to better capture late-onset toxicities and assess the long-term durability of local control, particularly in patients with prolonged survival.

## Conclusion

This study represents the largest frameless hf-GKRS study to date, specifically focused on treating treatment-naïve, large pf-METs. Our findings suggest that upfront hf-GKRS is a viable alternative for select patients, particularly those with larger treatment volumes who face an increased risk of toxicity or those who are at higher surgical risk.

## Data Availability

No datasets were generated or analysed during the current study.
